# Functional Outlook of *Penicillium digitatum PdMFS6* Transporter to Elucidate Its Role in Fungicide Resistance and Virulence

**DOI:** 10.3390/microorganisms13061213

**Published:** 2025-05-26

**Authors:** Paloma Sánchez-Torres

**Affiliations:** Food Biotechnology Department, Instituto de Agroquímica y Tecnología de Alimentos (IATA), Consejo Superior de Investigaciones Científicas (CSIC), Catedrático Agustín Escardino Benlloch 7, 46980 Paterna, Valencia, Spain; psanchez@iata.csic.es; Tel.: +34-963900022

**Keywords:** citrus, fungicide susceptibility, MDR, MFS transporter, *Penicillium digitatum*, postharvest, virulence

## Abstract

A novel *Penicillium digitatum* MFS transporter, PdMFS6 (PDIP_42530), was recognized, and its function was studied to explain its relevance in the simultaneous development of resistance to different fungicide spectrums. No changes were detected after application of chemical fungicides in mutants with the deleted gene, but chemical susceptibility was severely impaired in overexpressing strains, that became persistent to different chemicals. Furthermore, *P. digitatum* deleted transformants showed less fungal virulence appraise upon citrus infection stored at 20 °C. In strains derived from Pd149-*P. digitatum* with low virulence and overexpressing PdMFS6, the signs of the disease were more evident. In addition, evaluation of gene transcription showed an increase in PdMFS6 gene expression over time in all *P. digitatum* strains tested. It is noteworthy that during citrus fruit infection, the wild-type Pd1 strain displayed an augmented level of transcription, indicating that this transporter plays a role in infectivity. The fungal transporter PdMFS6 could contribute to the susceptibility to chemicals commonly used in postharvest treatments, as well as to rise the virulence of *P. digitatum* during fruit infection.

## 1. Introduction

Citrus fruits during postharvest can be affected by the pathogen *Penicillium digitatum* during storage and transportation, leading to green mold disease, which significantly reduces marketable citrus production [1,2]. The application of chemical fungicides to control this disease is usually the most widely used resource. However, their continued use has led to the emergence of resistance to these compounds. Efflux pumps are one of the mechanisms described in the development of resistance to chemical fungicides. Among these, the transporters of the Major Facilitator Superfamily (MFS) stand out [3].

Efflux transporters are essential membrane-bound proteins capable of transporting a wide variety of compounds, i.e., large molecules, particles, and minor elements, through biological barriers [4,5] and can generate defenses against an extensive compass of harmful products [6]. Efflux transporters, such as secondary MFS transporters, enable fungi to endure presence to harmful products by exporting compounds that may accumulate at toxic concentrations within fungal cells. Several studies have established a connection between increased efflux transporter function and the development of resistance to diverse types of fungicides in various pathogenic fungi [7,8,9], indicating a possible shared role of these transporters in determining fungicide sensitivity. MFS transporters are part of a very extensive, highly prolific group capable of transporting different products thanks to ionic gradients, that enables fungal growth in the presence of multiple drugs, a trait known as multidrug resistance (MDR). By transporting small molecules, they can regulate the growth of microorganisms under stressful circumstances by altering the membrane potential and internal pH [10]. In many fungal MFS transporters, there is a clear effect on toxic compounds outflow and fungicide resistance. In *Cercospora nicotianae* a MFS transporter played an important role in cercosporin autoresistance [11,12]. In *Botrytis cinerea*, sensitivity to camptothecin, cercosporin, as well as resistance to demethylation inhibitors (DMIs) fungicides, is mediated by the transporter MFS *BcMfs1* [13,14], and *mfsM2* was involved in the increase of fungicidal efflux activity [15]. Functional study in *MgMfs1* from *Mycosphaerella graminicola* demonstrated its role in particular sensitivity to strobilurins, but not to other fungicides tested [16,17]. *MfsA* from *Aspergillus carbonarius* appears to be involved in oxidative stress along with increased mycotoxin production [18]. A new mechanism of resistance to chemicals has been described in *Zymoseptoria tritici*, which causes the active expulsion of different unisite fungicides by the increase in the expression of the MFS1 gene [19]. In addition to its role in chemical sensitivity, the AaMFS19 transporter of the phytopathogenic fungus *Alternaria alternata* contributes to oxidative stress [20]. In *Penicillium expansum* strains with a multidrug-resistant phenotype, an induction of MFS transporter genes was detected based on transcriptomic analysis performed before and after contact to fludioxonil [21].

Genome analysis of *P. digitatum* has identified more than 90 MFSs [22]. Currently, only seven of the predicted MFSs, i.e., *PdMfs1*, *Pdmfs2*, *PdMFS1-5*, have been fully investigated [23,24,25,26]. Most of them are involved in sensitivity to different chemicals and, may also contribute to increased fungal aggressiveness. While *PdMfs1* only exerts sensitivity to imazalil, *Pdmfs2*, and *PdMFS1* are involved in resistance to prochloraz [23,24,25]. In addition, both are part of conidia production and disease progression pathways. Increased gene transcription of several MFS transporters in *P. digitatum* was demonstrated in a study evaluating the transcriptomic response after treatment with prochloraz [27]. *PdMFS1*, *PdMFS2*, and *PdMFS3* contribute to simultaneous resistance to some unconnected harmful products conferring multiresistant phenotype [25,26]. The characterization of both the genetic structure and size of the different MFS transporter proteins of *P. digitatum* showed their great diversity and that only a few of them were structurally included in the group of drug efflux pumps. However, those not included in this group were capable of conferring resistance to chemicals [26].

Given the ongoing interest in providing alternative options for fungal control, it is necessary to delve deeper into the factors attributable to resistance to chemical fungicides, which in many cases are related to the successful progression of pathogen infection due to increased virulence and ineffective control. Although MFS transporters have been previously described in *P. digitatum*, given the breadth and functional diversity of these transporters, this work addresses the role of a novel secondary transporter in *P. digitatum* (PdMFS6) through a functional analysis using gene deletion, over-expression in different genetic backgrounds, mutants’ characterization, and transcription studies to provide a better understanding of this family in order to select new targets for better control of the pathogen.

## 2. Materials and Methods

### 2.1. Strains and Culture Media

This work was performed using three *P. digitatum* strains: fungicide-resistant and highly virulent Pd1, and fungicide-sensitive Pd27 and Pd149, both highly virulent and very low virulent, respectively, as described previously [25]. For fungal development on axenic media and for conidia collection, potato dextrose agar (PDA; Liofilchem Laboratories, Waltham, MA, USA) or potato dextrose broth (PDB; Liofilchem Laboratories) was used, and conidia were collected as previously described [26]. Fungi were grown for 1 to 3 days at 25 °C in liquid cultures and for 1 week on a solid medium. The desired spore concentration was obtained from PDA plates counted using a hemocytometer. *Escherichia coli* DH5α was selected for cloning and vector production. *E. coli* was grown on LB medium containing 100 μg/mL kanamycin at 37 °C. *Agrobacterium tumefaciens* C_58_C_1_ was used for *P. digitatum* modification. Constructed plasmid were produced on LB medium with 50 μg/mL rifampicin and 100 μg/mL kanamycin at 28 °C as described [26].

### 2.2. Nucleic Acids Isolations

The total DNA of *P. digitatum* was obtained from mycelia as reported [10]. The purification of the obtained PCR fragments was carried out using the Ultra Clean™ PCR Clean-up system (MoBio, Solan Beach, CA, USA). The genetic modifications were confirmed by DNA sequencing [28]. The Trizol method (Ambion Inc., Austin, TX, USA) allowed the isolation of RNA from frozen mycelia of *P. digitatum*. Total RNA was extracted using fruit peel discs obtained during fruit infection [26].

### 2.3. Cloning and Characterization of PdMFS6

The identification of the genomic version of the PdMFS6 transporter of *P. digitatum* was carried out using the Pd1 DNA library and the oligos M6-1 and M6-2 (Table S1) [26]. PdMFS6 protein region evaluation was performed using SMART (http://smart.embl-heidelberg.de, accessed on 5 February 2025). The Clustal W program (https://www.genome.jp/tools-bin/clustalw, accessed on 7 February 2025) was used for protein alignments [29] and phylogenetic tree construction was done in Mega 7.0 program with the Maximum Likelihood method [30].

### 2.4. PdMFS6 Gene Elimination and Gene Over-Expression

For the elimination of the PdMFS6 gene, the upstream and downstream region flankings of the coding region were joined to the hph gene with the pRFHU2 vector [31]. For gene over-expression, the pRFHU plasmid was used [31], and the whole *PdMFS6* gene including promoter and terminator as described before [26].

C_58_C_1_ *Agrobacterium*-mediated transformation was performed as previously reported [26]. Selection media based on PDA with 100 μg/mL of hygromycin B was used for mutants screening. The validation of gene knock-out and gene over-expression was conducted by PCR and gene copy number was performed by qRT-PCR [26].

### 2.5. Infection Analyses

The fruits used in this study consisted of ripe oranges (*Citrus sinensis* L. Osbeck) of the ‘Navelina’ type without fungicide treatment, collected in the orchards at IVIA in Moncada (Valencia, Spain). For infection experiments, mature fruits were inoculated as previously described [25]. The infection tests were done twice and three replicates of five fruits each was carried out. Mock-inoculated fruits were used as a control. Infection development was evaluated as percentage of infected fruits (disease incidence) and diameter of macerated tissue (disease severity).

### 2.6. Chemical Sensitivity

The effect of different fungicides was determined by analyzing the antifungal activity using a 96-well microtiter plate growth assay, using 2.5 × 10^5^ conidia/mL. Four different chemical fungicides: Imazalil (IMZ) (Textar I; Tecnidex, Valencia, Spain), Prochloraz (PCL) (Ascurit; Tecnidex), Philabuster (PHI) (a mixture of Imazalil and Pyrimethanil) (Decco Ibérica, Paterna, Valencia) at 0, 0.5, 1, 2, 4, 8 and 10 µg/mL and Thiabendazole (TBZ) (Textar 60 T; Tecnidex) at 0. 5, 10, 20, 40, 80 and 100 µg/mL were used to test the sensitivity of *P. digitatum* strains. Fungal growth was assessed daily in the presence of increasing concentrations of chemicals for seven days relative to growth in the absence of chemicals. Growth was determined by OD at 492 nm in a Multiskan Spectrum microplate spectrophotometer (Thermo Electron Corporation, Vantaa, Finland). The growth medium was PDB (Liofilchen, Teramo, Italy) with 50 µg/mL of streptomycin (Sigma, Saint Louis, MO, USA) and the corresponding fungicide. Each treatment was performed in two independent experiments, each in triplicate. The sensitivity evaluation was expressed as percentage of growth as reported before [26].

### 2.7. Quantitative Real-Time PCR

First-strand cDNA synthesis was performed in 20 µL using the PrimeScript™ RT kit (Takara Bio Inc., Kusatsu, Japan). Quantitative PCR was performed as described [26]. Values were the average of three biological replicates with two replicates each. Oligos qM6F and qM6R were used in the reaction, and β-tubulin (qTubF-qTubR), 28S ribosomal protein (q28SF-q28SR), and histone H3 (qH3F-qH3R) were selected as independent reference genes (Table S1). Gene expression is calculated for PdMFS6 gene (GOI) relative to the reference gene (REF) using the modified equation EGOI^(−CqGOI)/EREF^(CqREF). LightCycler 480 SW 1.5 software (Roche Diagnostics, Basel, Switzerland) was used for cycle point quantification. Relative gene expression (RGE) was performed as previously described [26].

### 2.8. Statistical Analysis

The SAS statistical software version 15.3 (SAS 9.4) (SAS Institute Inc., Cary, NC, USA) was used for analysis of variance (ANOVA) to evaluate significant differences of data. Tukey’s mean separation test was used to determine statistically significant differences. The significant level for *p* values was considered as *p* < 0.05.

## 3. Results

### 3.1. Investigation of PdMFS6

*PdMFS6* was identified as a major facilitator superfamily transporter in *P. digitatum*. The genomic region contained an open reading frame with five introns and the deduced protein encodes 502 amino acids according to sequence evaluation. The complete sequence of the *PdMFS6* gene is available in GenBank as PDIP_42530 (Table 1).

**Figure 1:**
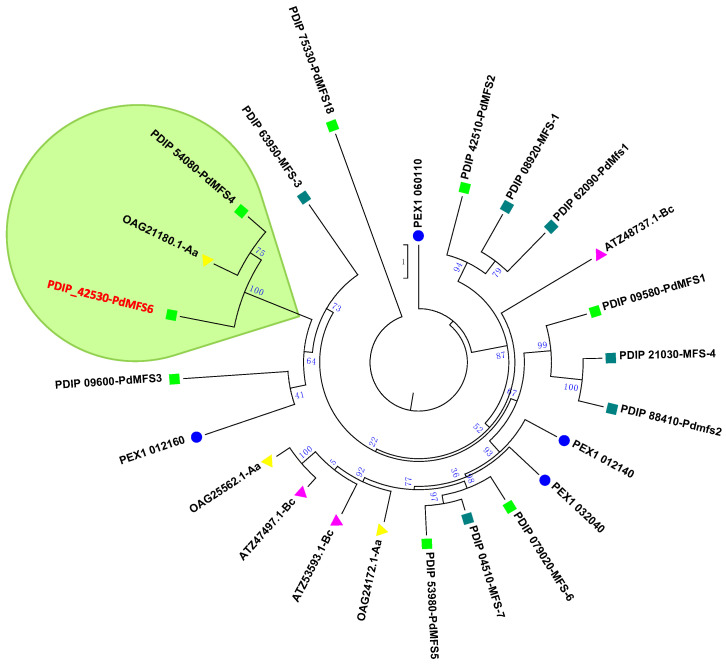
Phylogenetic tree of selected MFS transporters using unrooted Maximum Likelihood. PdMFS6 is highlighted in red. MFS transporters are represented by green squares (light green squares represent already characterized *P. digitatum* transporters, while dark green squares represent the remaining uncharacterized), blue circles (*P. expansum*), yellow triangles (*A. alternata*), and pink triangles (*B. cinerea*). The values indicate the number of times (in percentage) each branch topology was encountered during the bootstrap analysis. The names indicate the NCBI database accession number.

PdMFS6 was included as an MFS transporter because it presents a DNA binding region characteristic of this group. The software/TMPRED 3.0 (Swiss Institute of Bioinformatics) was used for hydropathic analysis that showed that PdMFS6 has 12 putative trans-membrane domains. Phylogenetic analysis of the PdMFS6 protein with other MFS transporters from *P. digitatum*, *P. expansum*, *B. cinerea*, and *A. alternata* available at the National Center for Biotechnology Information (NCBI) revealed that the PdMFS6 clusters close to *P. digitatum* MFS transporter PdMFS4 (PDIP_54080) [10] and OAG21180.1 MFS transporter of *A. alternata* as shown in the green framed area (Figure 1).

### 3.2. Characterization of Deletion and Over-Expression Mutants

**Figure 2:**
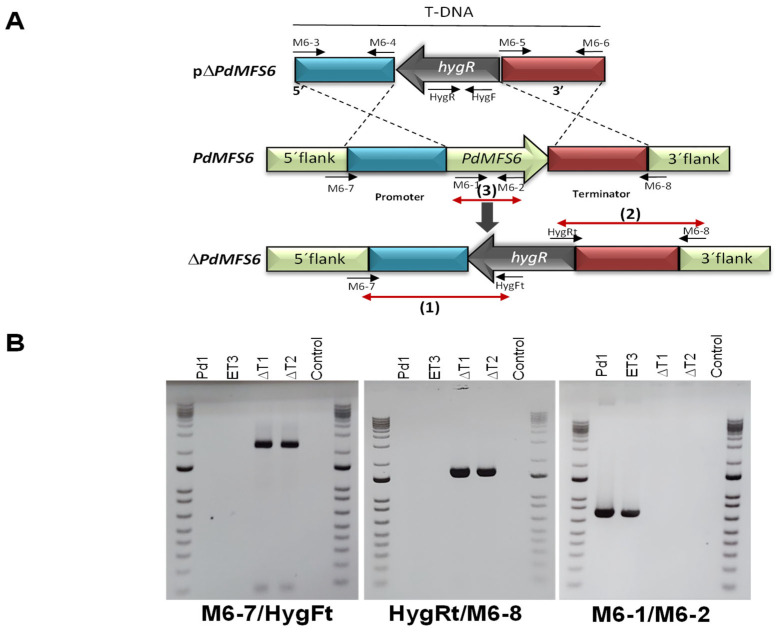
(**A**): Illustration of the wild-type locus and replacement of PdMFS6 with the selectable marker hygR from pΔPdMFS6 by homologous recombination to give rise to the ΔPdMFS6 transformants. (**B**): confirmation of gene deletion using the wild-type strain Pd1, two ΔPdMFS6 null mutants (ΔT1, ΔT2) and the respective ectopic mutant (ET3) as templates by polymerase chain reaction (PCR) with specific primers. Control corresponds to PCR reaction in the absence of template. (1): amplification from 5′ flank of each gene to *hph* gene, (2) amplification from 3′ flank of each gene to *hph* gene and (3) amplification of the coding region of the gene.

*A. tumefaciens* containing the vector pΔPdMFS6 (Figure 2A) was used to transform *P. digitatum* Pd1. The pΔPdMFS6 was built with a 1.5 kb fragment of the promoter gene region with the primers M6-3/M6-4, and a 1.6 kb fragment of the terminator gene region with the primers Mn-5/Mn-6 (Table S1), adjacent to the hph resistance cassette in the T-DNA region (Figure 2A). The target gene was validated with primers M6-7/HygFt in 5′ flank (promoter) and with primers HygRt/M6-8 in 3′ flank (terminator) (Table S1) (Figure 2B). The deletion of the coding region of the gene was confirmed by PCR with the oligos M6-1 and M6-2. The PCR bands only appeared in Pd1 and the ectopic transformant (ET3) and were missing in the deleted strains and negative control as expected (Figure 2B).

Plasmid pRFHU [31] was used to clone the complete PdMFS6 transporter gene (pOPdMFS6) containing a promoter (1.3 kb) and a terminator (1.0 kb) using oligos Mn-9/Mn-10 (Figure S1A). *P. digitatum* Pd27 and Pd149 strains were transformed using ATMT with pOPdMFS6 plasmid and three mutants of each parental were selected based on PCR validation with HygF/HygR. The expected 588-bp PCR fragment was perceived in all transformants and was missing from the untransformed Pd27 strain used as control (Figure S1B). T-DNA copy number evaluation was conducted by quantitative PCR using wild-type Pd27 and Pd149 strains as controls and the β-tubulin coding gene as an independent reference.

### 3.3. Fungicide Evaluation

**Figure 3:**
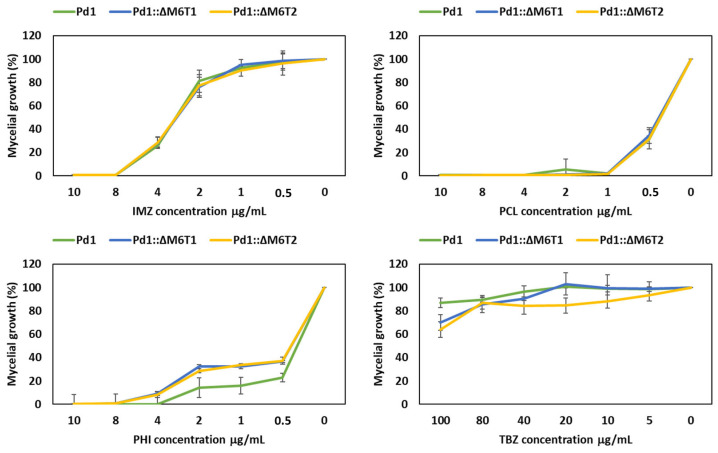
Assessment of chemical sensitivity after 7 days in deletant strains in contrast to wild type Pd1. The chemical concentrations are stated in μg/mL. Mycelial growth was considered with respect to each strain grown without fungicide. Error bars represent standard deviations of three replicates.

The growth of all selected *P. digitatum* strains (mutants and wild type) evaluated after 7 days at 25 °C on PDA amended with diverse fungicide concentrations displayed no significant variation in chemical sensitivity between strains lacking the *PdMFS6* gene and the wild-type parental strain Pd1 for IMZ, PCL, PHI and TBZ fungicides at all concentrations tested showing almost identical growth profile (Figure 3).

**Figure 4:**
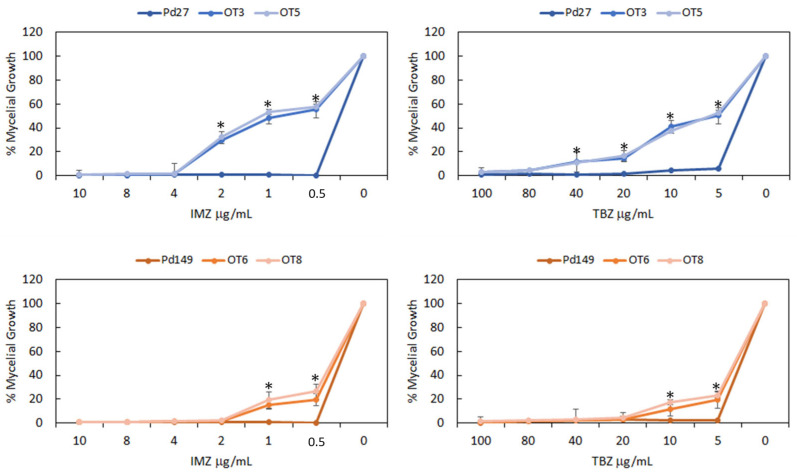
Evaluation of chemical sensitivity in over-expression Pd27-(OT3, OT5) strains and Pd149-over-expression (OT3, OT4) strains linked to respective wild types. The fungicides concentration is represented in μg/mL. Mycelial growth was calculated by comparing it with each strain grown without fungicide and expressed as a percentage. Error bars represent standard deviations of three replicates. * Significant differences between treatments using Tukey’s test (*p* < 0.05).

In over-expression mutants, only IMZ and TBZ were tested as representatives of two types of fungicides with different modes of action. All over-expression mutants, irrespective of the parental strain, presented a decreased susceptibility to the fungicides IMZ and TBZ. Pd27 mutants exhibited a higher level of resistance to fungicides than Pd149 mutants (Figure 4). The Pd149 mutants only maintained their resistance up to 1 µg/mL of IMZ, and the percentage of growth achieved was always lower than the increase displayed by the Pd27 mutants.

### 3.4. Infection Evaluation

**Figure 5:**
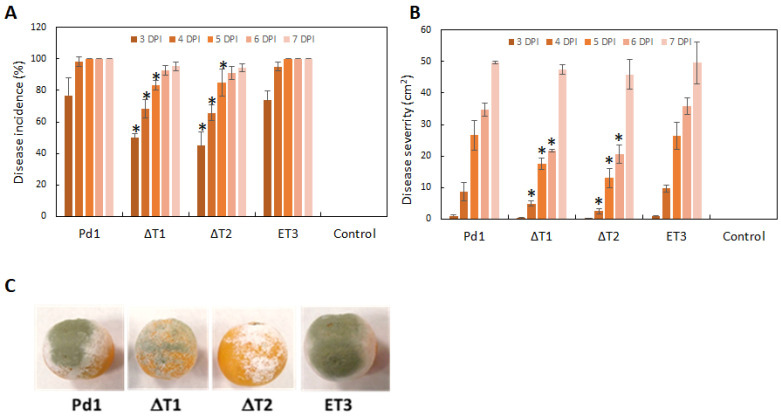
Infection assessment. (**A**): disease incidence (%) and (**B**): disease severity (cm^2^). Infection estimation of Pd1, ectopic transformant ET3 and knockout transformants (ΔT1, ΔT2). Results are expresses as mean of two experimental infections. Control related to oranges mock inoculated. Error bars represent standard deviations of three replicates * Significant changes between treatments using Tukey’s test (*p* < 0.05) at each dpi. (**C**): representative images of infected orange fruits at 6 dpi.

All fungi used in this work were assessed, with disease incidence meaning a percentage of infected fruit and disease severity meaning macerated surface area after several days post-infection (dpi). Infection of the ectopic *P. digitatum* strain ET3 behaved identically to the parental strain Pd1. However, deletion of the *PdMFS6* gene in both deletants (ΔT1 and ΔT2) occasioned a reduction in virulence, mainly through the early stages (3–5 dpi), as shown by the value of rot incidence (Figure 5A). The effect on disease severity was consistent with that detected for incidence, although the decrease extended between 4 and 6 dpi (Figure 5B,C). The average reduction in virulence in the deletion mutants resulted in a 25% to 35% reduction in both rot incidence and maceration. No significant alterations in infectivity were displayed at 7 dpi.

**Figure 6:**
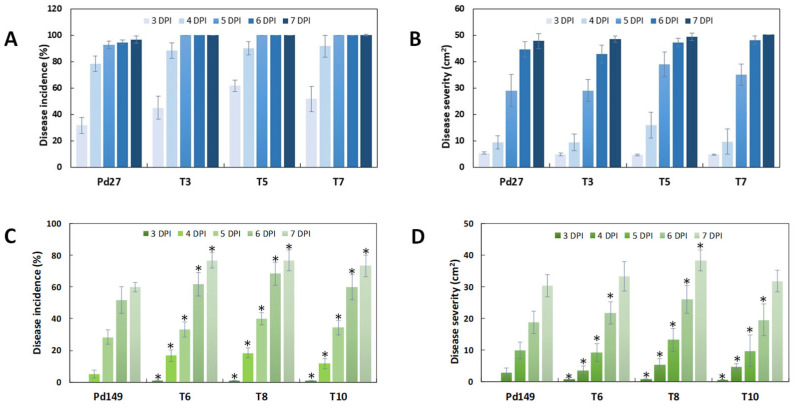
Disease incidence (%) (**A**,**C**) and disease severity (cm^2^) (**B**,**D**). Virulence estimation of Pd27-over-expression strains (T3, T5, T7) and Pd149-over-expression strains (T6, T8, T10). Results are represented as mean of three infection experiments. Oranges mock inoculated represented the control. Error bars signify standard deviation. * Significant differences between treatments using Tukey’s test (*p* < 0.05) at each dpi.

Infection examination was also achieved for over-expression strains. Pd27-transformants (OT3, OT5, and OT7) did not display significant changes when related to parental strain Pd27 and fungal strains acted similarly in disease incidence and severity (Figure 6A,B). In contrast, Pd149 strains (OT6, OT8, and OT10) showed an increase in virulence in both disease incidence and severity, although the disease levels reached were not comparable to the damage caused by Pd1 (Figure 6).

### 3.5. Transcription Profile of PdMFS6

**Figure 7:**
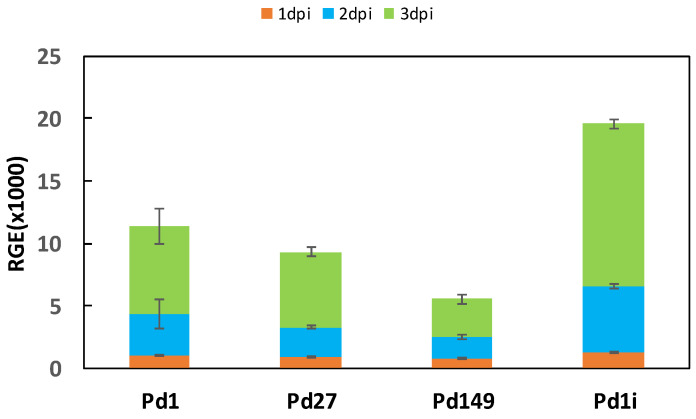
Examination of *PdMFS6* comparative gene transcription (RGE). Temporal evaluation of gene expression of Pd1, Pd27 and Pd149 in axenic growth at 25 °C and Pd1 infecting orange (Pd1i). Orange, blue and green colors link to 1 dpi, 2 dpi, and 3 dpi, respectively. Error bars represent standard deviations of three biological repeats.

Expression analysis of *PdMFS6* gene in the different parental strains tested showed that the highest level corresponded to Pd1, followed by Pd27 and finally Pd149. The expression profile showed an increase over time in all three strains, with the highest transcription peak at 3 dpi. Evaluation of *PdMFS6* gene expression during infection of the Pd1 strain in oranges showed induction of transcription at all times tested, reaching a gene transcription rate twofold higher at 3 dpi (Figure 7).

## 4. Discussion

MFS transporters constitute one of the largest active secondary translocators family and are widely distributed and conserved across all kingdoms (animals, plants and fungi) [32,33,34]. Currently, they are key players in toxicant removal or sugar transport and are increasingly recognized for their involvement in a wider range of physiological functions [35,36,37]. Despite the large number of MFS transporters identified in the genomes of fungal phytopathogens [3], the specific functions of many of them remain unknown due to the high number of members that are part of the extensive MFS transporter family. Therefore, in this study, we characterize PdMFS6, a novel MFS transporter from *P. digitatum*.

PdMFS6 with 502 amino acids falls into the majority of MFS proteins consisting of 400–600 amino acid residues not highly conserved since exhibited a sequence protein similarity of 12 to 18% [38]. As expected, MFS proteins from different subfamilies exhibit significant sequence divergence. Phylogenetic analysis of MFS fungal transporters from various plant pathogens reveals that many cluster into distinct groups, and orthologs are not consistently present across different fungal species [39]. In this work, the phylogenetic analysis included different MFS transporters from several phytopathogenic species such as *P. expansum*, *B. cinerea*, *A. alternata* and other previously described *P. digitatum* transporters. PdMFS6 clustered with the group composed of a transporter from *A. alternata* and PdMFS4 from *P. digitatum* showed a clear role in fungal virulence but lacks the ability to confer resistance to fungicides [26]. These features are similar to those found in PdMFS6, although with some differences.

Studies related to resistance to chemical fungicides demonstrated that by eliminating the PdMFS6 transporter, the susceptibility profile to those chemicals was not modified, in the same way as occurred with the PdMFS4 and PdMFS5 transporters of *P. digitatum* [26]. However, when over-expression mutants were evaluated in two genetic backgrounds, a modification in sensitivity to fungicides such as IMZ and TBZ, frequently used in postharvest citrus, was observed. The results for PdMFS6 were contrary to those reported for other *P. digitatum* transporters (PdMFS4 and PdMFS5), whose sensitivity to fungicides was not modified by either gene deletion or over-expression [26]. On the contrary, the *P. digitatum* transporter PdSUT1 showed a similar effect to PdMFS6 by indirectly contributing to chemical sensitivity, since although PdSUT1 deletants did not modify its sensitivity to fungicides, the over-expression mutants did [40]. One possible explanation could be that PdMFS6′s function is not related to the efflux of fungicides, but to other types of molecules. Therefore, its elimination does not affect its sensitivity, but over-expression in sensitive strains could force the pumping of toxic products. In fact, over-expression of MFS has been shown to facilitate the movement of small molecules across cellular barriers in response to chemical imbalance [41]. Alternatively, the function performed by PdMFS6 could be replaced by other *P. digitatum* MFS transporters when this transporter is eliminated due to the numerous members that form part of this large family.

On the other hand, MFS transporters have been linked to effector molecules that could be responsible of the infectivity of fungi [42,43,44]. Knockdown of *PdMFS6* confirmed its role in virulence, as suggested by a reduction in green mold incidence and severity. Likewise, mutants overexpressing Pd149-PdMFS6 had increased infectivity, confirming the contribution of the PdMFS6 gene to virulence. The increase in virulence was not detected in Pd27-overexpressing transformants, probably because their virulence level was already high from the start, and the increase in virulence is not easily detectable. The same result has already been described in other *P. digitatum* genes, such as PdSUT1 [40] and PdMFS1 [25].

Previous work showed that elimination of different MFS transporters from *P. digitatum* reduced its virulence [24,25,26]. The relevant role of PdMFS6 in fungal infectivity could be related to the elimination of host protection products. Earlier studies showed that some transporters play an important function in aggressiveness such as the *B. cinerea* transporter mfsG in *Arabidopsis thaliana* [33], FgMFS1 in *Fusarium graminearum*, which is crucial for pathogenicity in wheat [42].

The contribution of PdMFS6 to fungal infection was also confirmed by assessing gene expression. During in vitro growth, MFS transporters showed an increasing profile over time in all three strains analyzed, with Pd1 presenting the highest transcription rate, as occurs with other *P. digitatum* transporters, i.e., PdMFS2-PdMFS5 [26], with the exception of what was observed with PdMFS1 of *P. digitatum*, which showed the same gene transcription rate in all wild-type strains [25]. Despite all this, the role of MFS transporters in infectivity remains unclear. Previous works showed that five (PdMFS1-PdMFS5) *P. digitatum* MFS transporters contributed to a greater or lesser extent to fungal virulence [25,26]. Moreover, while its participation in virulence has been demonstrated in cases such as the Ctb4 transporter in *C. nicotianae* [43] or the AaMFS19 transporter in *A. alternata* [17], this has not been the case for the BcMfs1 transporter in *B. cinerea* or MgMfs1 in *M. graminicola* [16,45]. In this study, *P. digitatum* Pd1 showed that the transcription rate increased significantly in the late stages of infection when compared to axenic growth confirming its function in the infectious capacity.

In general, the mechanisms regulating MFS transporters are poorly understood. It is thought that the transcription of transporter genes could be controlled by fungal transcription factors belonging to the zinc type (Zn_2_Cys_6_) [46]. In *A. alternata*, MFS transporter *AaMFS19* is regulated by the transcription activators Yap1 and the mitogen-activated protein kinases (MAPKs) Fus3 and Hog1 [20]. For yeasts, MFS transporters are regulated through different stress-related TFs, such as Yap1, Msn2, Msn4, and Sfp1 [10]. In *PdMFS6*, the TF *PdSte12* and the MAPK *PdSlt2* appear to act as negative regulators in the same way as described for some of the characterized *P. digitatum* MFS [47,48]. Earlier studies with fungal transporters and their transcriptional inducers have shown that they are involved in numerous processes to enhance the fight against harmful agents [49]. It remains unclear whether a general regulatory mechanism controls the activation or repression of MFS transporters in *P. digitatum*, highlighting the need for further investigation.

## 5. Conclusions

This work has allowed us to explore deeper into the diverse functions attributable to the extensive family of MFS transporters in the context of the interaction between *P. digitatum* and citrus fruits. Based on our results, we can conclude that *PdMFS6,* although presenting a low level of gene transcription, could contribute to the acquisition of MDR phenotypes and plays a relevant role during citrus fruit infection. The mechanisms involved in both functions could underlie the transport of different molecules (nutrients), which will require further research in the future.

## Figures and Tables

**Table 1:** Features of *PdMFS6* gene.

Gene Identification	PDIP_42530
Gene size (bp)	**1727**
N° Introns	**5**
Transmembrane helices	**12**
Protein Size (aa)	**502**

## Data Availability

The original contributions presented in this study are included in the article/Supplementary Materials. Further inquiries can be directed to the corresponding author.

## Supplementary Materials

The following supporting information can be downloaded at: https://www.mdpi.com/article/10.3390/microorganisms13061213/s1, Figure S1: over-expression; Table S1: oligonucleotides.

## Abbreviations

The following abbreviations are used in this manuscript:

ATMT	*Agrobacterium tumefaciens* mediated transformation
MFS	Major Facilitator Superfamily
MDR	Multi Drug Resistance
DMI	Demethylation inhibitor
TF	Transcription Factor
MAPK	mitogen-activated protein kinase
IMZ	Imazalil
TBZ	Thiabendazole
PHI	Philabuster
PCL	Prochloraz
DPI	Days Post Infection
