# Supplementary material for: Functional Outlook of Penicillium digitatum PdMFS6 Transporter to Elucidate Its Role in Fungicide Resistance and Virulence
Source: Microorganisms. 2025 May 26;13(6):1213. doi: 10.3390/microorganisms13061213 (PMC12194951; doi:10.3390/microorganisms13061213)
Supplement: Supplementary file 1 [file microorganisms-13-01213-s001.zip › microorganisms-3604840-supplementary.pdf]

Table S1. Oligo sequence used in this study.

Name	Sequence (5'-3')
M6-1	ATGGTATCGTCAAAGACTGG
M6-2	AGAAGAAAAGACTGGTACG
M6-3	GGTCTTAAUTGAATTAGTGGATGGGCTTGTC
M6-4	GGCATTAAUGAAGTAATGATCTGCTCGCC
M6-5	GGACTTAAUACATTCCAGTCGAGGGCTTG
M6-6	GGGTTTAAUTCGGTGGAAGACATAATCG
M6-7	GGTCTTAAUACGAGGAGCTGCTAGGCCGC
M6-8	ACGTCATTCCACCCCGATCATG
M6-9	GGGTTTAAUTGAATTAGTGGATGGGCTTGTC
M6-10	GGACTTAAUTCGGTGGAAGACATAATCG
HygRt	ATCGAAGCTGAAAGCACGAG
HygFt	GGCAATTTCGATGATGCAGC
HygR	AGCTGCGCCGATGGTTTCTACAA
HygF	GCGCGTCTGCTGCTCCATACAA
hTubF	AGCGGTGACAAGTACGTTCC
hTubR	ACCCTTAGCCCAGTTGTTAC
qTubF	AGCGGTGACAAGTACGTTCC
qTubR	ACCCTTAGCCCAGTTGTTAC
q28SF	TTATAGCCGAGGGTGCAATG
q28SR	TTTCAAGACGGGTCGCTTAC
qH3F	AGGCTCCCCGTAAGCAGCTCGC
qH3R	CGACATGAGGCGGAACTTACCGG
qM6-F	ATCACAACTGCTCTCCGCG
qM6-R	ACTTTACAAAGGCCTGGAAG

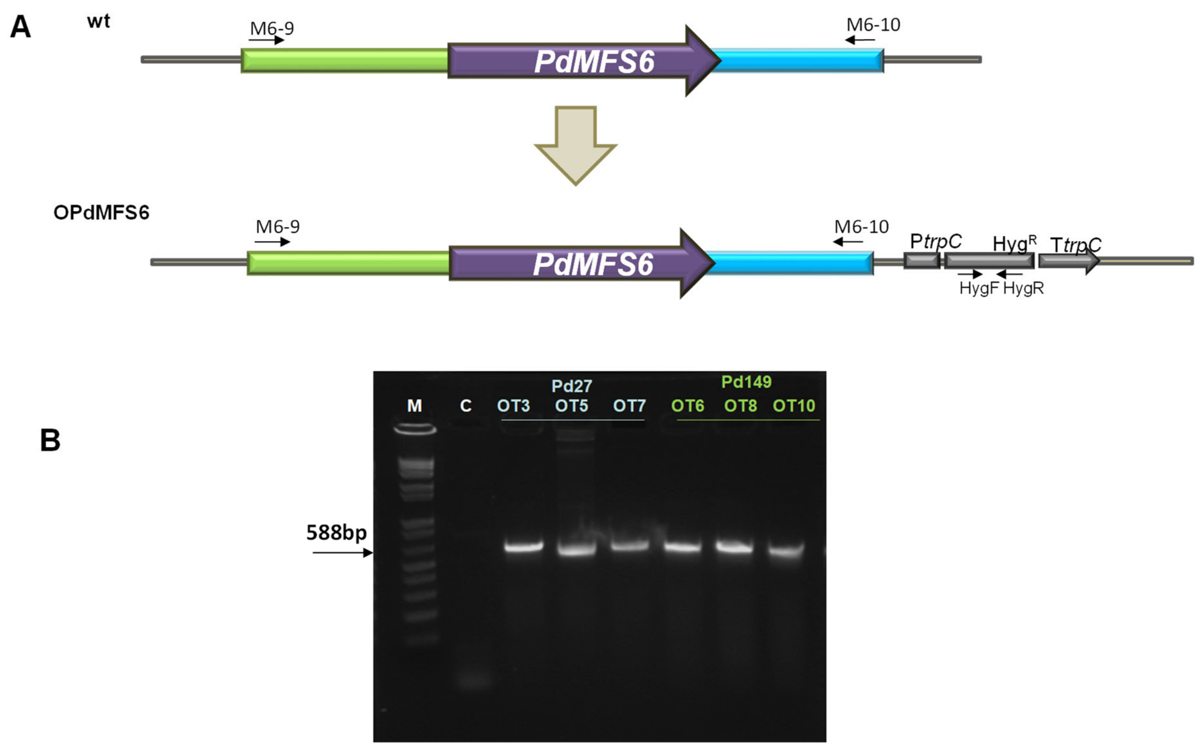

Figure S1. Construction and confirmation of *P. digitatum* overexpression *PdMFS6* transformants. (**A**) Map of plasmid pOMFS6. Diagram of wild-type locus and the *PdMFS6* gene with the HygR selectable marker from pOMFS1 inserted elsewhere in the genome. Primers used in the construction of plasmid pOMFS6 and those used for the analysis of the transformants are shown. (**B**) Hygromycin polymerase chain reaction (PCR) analysis of the control Pd27 wild type and Pd27-transformants and Pd149-transformants using primers HygF/HygR.
